# Rydberg polaritons in ReS_2_ crystals

**DOI:** 10.1126/sciadv.add8857

**Published:** 2022-11-23

**Authors:** Annalisa Coriolano, Laura Polimeno, Marco Pugliese, Alessandro Cannavale, Dimitrios Trypogeorgos, Anna Di Renzo, Vincenzo Ardizzone, Aurora Rizzo, Dario Ballarini, Giuseppe Gigli, Vincenzo Maiorano, Adzilah Shahna Rosyadi, Ching-An Chuang, Ching-Hwa Ho, Luisa De Marco, Daniele Sanvitto, Milena De Giorgi

**Affiliations:** ^1^ CNR-NANOTEC, Institute of Nanotechnology, Via Monteroni, Lecce 73100, Italy.; ^2^ Dipartimento di Matematica e Fisica E. De Giorgi, Università del Salento, Campus Ecotekne, Via Monteroni, Lecce 73100, Italy.; ^3^ Department of Civil Engineering Sciences and Architecture, Polytechnic University of Bari, Bari, Italy.; ^4^ Graduate Institute of Applied Science and Technology, National Taiwan University of Science and Technology, Taipei 106, Taiwan.

## Abstract

Rhenium disulfide belongs to group VII transition metal dichalcogenides (TMDs) with attractive properties such as exceptionally high refractive index and remarkable oscillator strength, large in-plane birefringence, and good chemical stability. Unlike most other TMDs, the peculiar optical properties of rhenium disulfide persist from bulk to the monolayer, making this material potentially suitable for applications in optical devices. In this work, we demonstrate with unprecedented clarity the strong coupling between cavity modes and excited states, which results in a strong polariton interaction, showing the interest of these materials as a solid-state counterpart of Rydberg atomic systems. Moreover, we definitively clarify the nature of important spectral features, shedding light on some controversial aspects or incomplete interpretations and demonstrating that their origin is due to the interesting combination of the very high refractive index and the large oscillator strength expressed by these TMDs.

## Teaser

The strong coupling between light and Rydberg excitons in ReS2 crystals results in large polaritonic interactions.

## INTRODUCTION

Transition metal dichalcogenides (TMDs) with chemical formula MX_2_ (e.g., M = Mo, W, Re; X = S, Se) are van der Waals materials with outstanding structural and optical properties, such as chemical stability (*1*), mechanical flexibility (*1*), high binding energies (*2*, *3*) and oscillator strengths, and narrow photoluminescence linewidths (*4*), which make them extremely attractive in a plethora of photonics and optoelectronics applications (*5*–*7*). Their optical and chemical properties vary according to which group the TMDs belong.

In particular, group VI TMDs—such as MoS_2_, MoSe_2_, WS_2_, and WSe_2_—are characterized by linear isotropic in-plane optical properties due to the high symmetry of their crystal structure. Moreover, they show a transition from indirect to direct bandgap when going from bulk to a monolayer. This is due to their strong interlayer coupling, which is broken when the out-of-plane confinement is achieved with single layers (*8*).

**Fig. 1.**
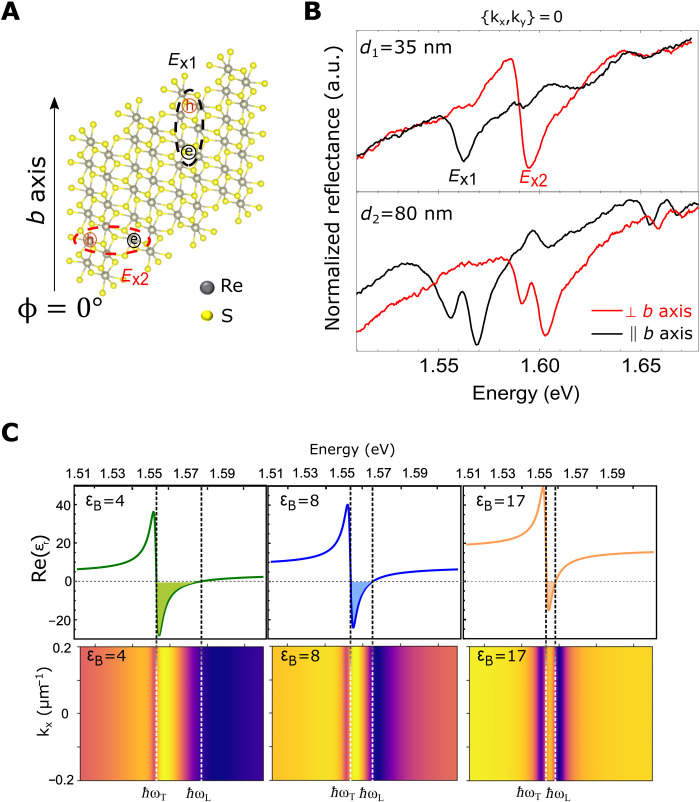
Origin of exciton splitting in reflection spectra. (**A**) Sketch of ReS_2_ monolayer atomic arrangement. The Re4 clustering chains, *b* axis, and the dipole direction of the main excitons are also indicated. The angle ϕ is defined as the polarization angle with respect to the *b* axis. (**B**) Reflection spectra of a 35-nm-thick (top) and an 80-nm-thick (bottom) ReS_2_ crystal exfoliated on a glass substrate for parallel (black line) and perpendicular (red line) polarization respect to the *b* axis. a.u., arbitrary units. (**C**) Top panels show how different values of the real part background permittivity, ε_B_, affect the reflectivity spectra close to the exciton resonance. Note that left and right panels reproduce the effect of a low and high permittivity on materials with a high oscillator strength, such as perovskites and ReS_2_, respectively.

On the other hand, group VII TMDs, such as ReS_2_ and ReSe_2_, crystallize in a distorted single-layer trigonal (1T′) structure of triclinic symmetry (Fig. 1A) due to the Re-Re interaction aligned along the *b* axis. This results in reduced crystal symmetry, which leads to strong in-plane anisotropic optical properties (*9*, *10*), inducing the formation of two almost orthogonally polarized in-plane excitons (*9*) and high optical birefringence (*11*, *12*). These properties are exploited for different applications, such as field effect transistors (*13*, *14*), polarized photodetectors (*13*, *15*), and photocatalyst (*16*). Unlike other TMDs, ReS_2_ and ReSe_2_ are also characterized by a direct bandgap that persists from bulk to monolayer due to the distorted 1T structure (*17*) that hinders ordered stacking of neighboring layers and minimizes the interlayer overlap of wave functions, as shown by density functional theory calculations (*18*). Such a weak interlayer coupling makes it possible to achieve the same properties as two-dimensional (2D) systems, regardless of the number of layers, avoiding the challenging and time-consuming preparation of large-area monolayers. In addition, they have a very high refractive index in the visible/near-infrared spectral region, a quite unique feature compared to other materials (*19*). This makes ReS_2_ extremely interesting for photonic applications and a unique platform for the exploration of novel topological properties when used as metamaterials.

Materials with planar optical anisotropy that support matter-light quasiparticles (i.e., polaritons), resulting from the strong coupling between excitons and photons, are extremely interesting thanks to their potential for the realization of topological exciton-polariton systems. This is mainly due to the possibility of easily tuning the optoelectronic properties of the polariton device by actively changing different parameters, such as crystal thickness, polarization, external magnetic and electric field (*20*), and sample temperature. Moreover, it has been theoretically predicted that exciton-polariton condensates can exhibit longer-range algebraic correlations under nonequilibrium conditions only in strongly anisotropic systems (*21*). All these reasons make the highly anisotropic ReS_2_ crystals, as active materials in exciton-polariton systems, very intriguing (*22*, *23*).

In this work, we unambiguously demonstrate the polarization-dependent strong coupling in ReS_2_ crystal and the hybridization between different higher-order exciton states resulting in Rydberg polaritons with enhanced interactions, making this material useful for the realization of polarization-controlled polaritonic devices. Taking advantage of the spectral features of strongly coupled ReS_2_ polaritons, we demonstrate that ReS_2_ crystals have only two orthogonally polarized excitons confuting previous studies, suggesting four excitonic resonances (*24*, *25*).

## RESULTS AND DISCUSSION

The ReS_2_ crystal structure is shown in Fig. 1A: Each crystal layer of Re atoms is placed between two S sheets, with distorted trigonal antiprismatic coordination and strong covalent bonding between the Re and S atoms. Rhenium atoms (gray) form a chain due to the Re-Re bonds, which defines the *b* axis of the crystal. Because of the strong metal-metal bond, ReS_2_ breaks preferentially along the *b* axis (*26*, *27*), typically forming a longer crystal edge after mechanical exfoliation.

The distortion of the ReS_2_ atomic structure induces the strong anisotropy of the exciton resonances, resulting in a different orientations and optical selection rules for linearly polarized light (*25*, *28*, *29*). The typical polarized reflection spectrum for a 35-nm-thick ReS_2_ crystal exfoliated on glass substrate (top layer of Fig. 1B) are mainly characterized by one exciton resonance, *E*_x1_, polarized parallel to the *b* axis (ϕ = 0°) (black line) and a second exciton resonance, *E*_x2_, polarized almost perpendicular to the *b* axis (ϕ = 90°) (red line). Moving to thicker crystals, we observe some changes in the reflection spectra: 80-nm-thick ReS_2_ exhibits an exciton resonance splitting for each polarization, and excited state transitions appear at higher energy (~1.65 eV) as shown in the bottom layer of Fig. 1B.

Additional resonances around the main exciton have already been observed in previous works (*22*, *23*), and, as in our case, these features have been distinguishable for crystals exfoliated in thick flakes (≥50 nm) (*9*, *25*), whereas for the thinner one, only the main exciton transition is distinctly observable (*9*, *29*–*31*). However, their nature is still debated. In the work of Arora *et al.* (*25*), multiple close-lying bright excitons were associated to degenerate direct transitions twofold from the valence band maximum to the conduction band minimum, with each degenerate pair consisting of bands with opposite spins. Additional peaks appearing below the exciton in thick crystals have been attributed to donor bound excitons (*9*), whereas Dhara and colleagues (*23*, *24*) hypothesized that these peaks are (i) due to the splitting of singlet and triplet states of excitons as a result of the electron-hole exchange interaction or (ii) induced from the broken rotational symmetry due to the structural anisotropy and spin-orbit coupling of ReS_2_. Recently, Gogna *et al.* (*22*) hint at an apparent splitting of the exciton resonance due to the cavity effect caused by reflections within the flake. Here, we assess that the two resonances that appear for both polarized excitonic transitions are, in fact, the longitudinal and transverse excitons to which the polaritonic branches asymptotically tend and observable in ReS_2_ crystals for flakes thicker than 50 nm. This is due to the unique combination of material optical parameters, mainly (i) the very high background refractive index and (ii) the large oscillator strength associated to the ReS_2_ excitons.

To investigate the role of the high background refractive index on the reflectivity of the ReS_2_, we consider the reflectance, at zero-order approximation, given by $R=\frac{(1-n{)}^{2}+k^{2}}{(1+n{)}^{2}+k^{2}}$, where *n* and n' are the real and imaginary part of the complex refractive index, respectively. Because of the very high real part of the refractive index, n=Re[ϵ(ω)]>4 (where ε(ω) is the complex permittivity), the crystal flakes, when lying on a low index material, behave as a dielectric slab resonator supporting Fabry-Perot modes. Because of the high oscillator strength, *f* ∼ 0.3 eV^2^, of the excitonic transitions, there is a strong interaction between these modes and the exciton resonances, giving rise to new hybrid states called polaritons (*32*).

This can be easily described by modeling the two polarization-dependent exciton resonances, *E*_X1_ and *E*_X2_, with a Lorentz oscillator using a dielectric function given by

$$\varepsilon_{1,2}(\omega )=\varepsilon_{B_{1,2}}+\frac{\;f_{1,2}}{E_{x_{1,2}}^{2}-E^{2}-iE\Gamma_{1,2}}$$ (1)

where *E* = ħω, ε_B_ is the background permittivity, *E*_X_ is the excitonic resonance, Γ is the exciton linewidth, and *f* is the oscillator strength. We found that in ReS_2_, because of the peculiar combination of high refractive index and strong exciton oscillator strength, the real part of the permittivity crosses zero and becomes negative around the exciton resonance (Fig. 1C, top right). By considering the spatial evolution of the field in this negative epsilon region (negative permittivity), following Maxwell’s equations (*33*), we can obtain two class of solutions given by (i) ε (ω) = 0 and (ii) $\vec{k}∙\vec{E_{\mathrm{ElectricField}}}=0$. The first solution Ε_L_ = ħω_L_ corresponds to the appearing of a longitudinal mode, which is usually invisible in ordinary materials, while the second solution *E*_T_ = ħω_T_ corresponds to the standard transversal mode (*34*, *35*). As a result of the appearance of both self-hybridized modes in the region with Re[ε] < 0, the electromagnetic wave cannot propagate into the material but rather exponentially decay, resulting in a strong effective reflectivity (*36*). Note that the energy position of the longitudinal mode and the gap between *E*_L_ and *E*_T_ strongly depend on the background permittivity of the material, ε_B._ By decreasing ε_B_, the longitudinal modes shift at higher energy, resulting in an increase in the longitudinal-transversal energy splitting Δ*E*_L/T_ = ħω_L_ − ħω_T._ (Fig. 1C, top) but with a smoother transition between the region with Re[ε] < 0 and Re[ε] > 0. On the other hand, for fixed background permittivity, ε_B_, Δ*E*_L/T_ increases for higher coupling strength between the exciton and photons (i.e., higher oscillator strength of the exciton resonance, fig.S1). It is therefore clear that both modes can be sharply seen only in those materials that have a high permittivity while keeping an equally high oscillator strength. ReS_2_ has the chance to meet both criteria.

The polarized reflectance spectrum of ReS_2_ crystals exfoliated on top of a glass substrate has therefore this characteristic. Simulations using the semianalytical rigorous coupled-wave analysis method (*37*) depict very well this behavior. Note that, looking at Fig. 1C, materials such as perovskites (parameters simulated in the bottom left panel) with a high background refractive index (*n* ∼ 2, i.e., ε_B_ ∼ 4) and a real part of the permittivity that also becomes negative do not show two sharp resonances due to the smooth variation of the permittivity if compared to the ReS_2_, which is simulated in the righthand side of Fig. 1C.

**Fig. 2.**
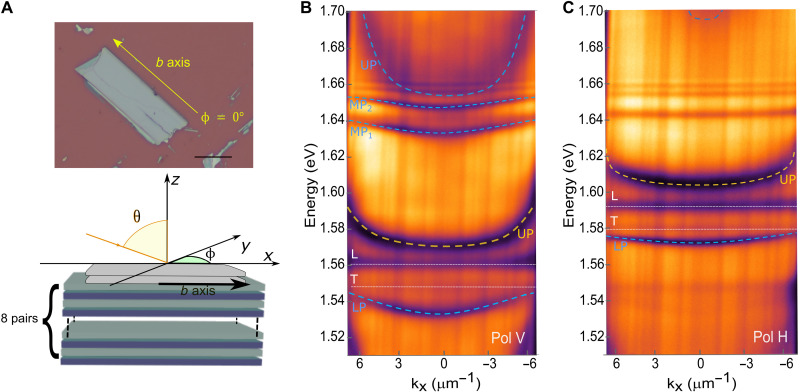
Strong coupling with Rydberg excitons. (**A**) Top: Optical microscope image of a 310-nm-thick ReS_2_ flake exfoliated on a DBR. Scale bar, 20 μm. Bottom: Scheme of the structure composed of a bottom DBR made by eight SiO_2_/TiO_2_ pairs with a ReS_2_ crystal (gray) on top. (**B** and **C**) Energy versus k_x_ in-plane momentum reflection spectra polarized parallel to the *b* axis (B) and perpendicular to the *b* axis (C). The blue dashed lines are the calculated polariton branches resulting from the strong coupling of the exciton with a cavity mode: LP is the lower polariton, whereas the MP_1,2_ are the first and second middle polaritons; the yellow dashed line is the polariton upper polariton (UP) branch induced by the coupling of the exciton with the previous mode, whereas the white dashed lines are the energies of the transversal (T) and longitudinal (L) modes.

In the following, we exploit the high refractive index and the strong oscillator strength of the excitonic transitions in ReS_2_ to investigate the full hybridized dispersion of the ground-state excitons and the first two excited states under strong coupling regime. To do so, we have exfoliated a 310-nm-thick ReS_2_ crystal (Fig. 2A and fig. S3) on a distributed Bragg reflector (DBR) that increase the mode finesse without reducing the photoluminescence collection from the front side of the crystal. Figure 2 (B and C) shows the linearly polarized energy reflection as function of the in-plane momentum, k_x_. The spectra unambiguously evidence the typical dispersions of a system in strong light-matter coupling regime, with the folding of the energy bands for the two excitons depending on the direction of polarization.

Because of the strong in-plane optical birefringence present in ReS_2_ crystal, the real part of the refractive index along the Re-Re chain direction (*b* axis, ϕ = 0°, *n* = 4.1) is ∼20% higher than the ones along the perpendicular direction (ϕ = 90°, *n* = 3.2) (*11*, *38*, *39*). Consequently, the photonic modes of the structure shift at higher energy for the H polarization with respect to the V polarization. This results in a different detuning of the polariton states with a different fraction of photon and exciton for each polaritonic band in the two linear polarizations.

A detailed theoretical analysis of the reflectivity at {k_x_, k_y_}= 0 as a function of the crystal thickness has allowed to associate the various dispersion curves for the two polarizations. For the V polarization, both the exciton *E*_X1_ and the excited states are strongly coupled to a Fabry-Perot photonic mode (fig. S4A), resulting in the formation of lower polariton (LP, at 1.533 eV), middle polaritons (MP_1_, at 1.632 eV and MP_2_ at 1.647 eV), and upper polariton (UP, at 1.653 eV) branches (dashed blue guidelines and label in Fig. 2B). The polariton state evidenced by the dashed yellow line instead represents the upper polariton branch, generated by the strong coupling of the exciton *E*_X1_ with a previous Fabry-Perot photonic mode (energy of the bare mode ~ 1.4 eV). The corresponding lower polariton branch of this other Fabry-Perot mode is not experimentally observable because its energy is outside the stopband of the DBR. Last, the transitions evidenced by the pointed dashed white lines are the transversal and longitudinal modes as described in Fig. 1B and 1C.

**Fig. 3.**
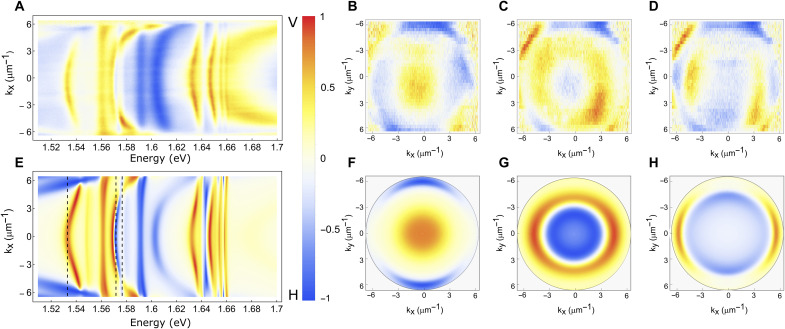
Degree of polarization in k space. (**A** to **H**) Experimental (A) and theoretical (E) degree of linear polarization (DOP) of the reflectance signal in the horizontal/vertical (H/V) basis. Experimental (B to D) and theoretical (F to H) DOP of the reflectance maps in the momentum space at isoenergetic cross sections corresponding to the lower polariton of the exciton *E*_X1_ (B and F), the lower polariton of the exciton *E*_X2_ (C and G), and at the energy where the lower polariton of exciton *E*_X2_ that crosses at the higher k_x_ values the upper polariton of *E*_X1_ (D and H).

The dispersion spectrum in the H-polarized direction (Fig. 3C) shows a similar behavior for the exciton *E*_x2_ despite a different detuning of the mode and lower oscillator strength. However, the excited states remain uncoupled being the photonic modes highly detuned from these energies (see also fig. S3B). By changing the ReS_2_ crystal thickness, we can also be able to tune the coupling of the exciton and the one of the excited states, varying the number and position of the photonic modes (figs. S5 and S6).

By making a 1D raster scan, we can reconstruct the reflectance spectra in both directions of the Fourier plane. The k_x_-k_y_ dispersion maps for the two polarizations are plotted in Fig. 3A in terms of the degree of linear polarization (DOP), defined as (*I*_V_ − *I*_H_)/(*I*_V_ + *I*_H_), where *I*_H_ and *I*_V_ are the reflected light intensities for horizontal and vertical polarizations, respectively. Figure 3 (A and E) demonstrates the good agreement between the experimental DOP (Fig. 3A) along the **k_x_** direction at **k_y_** = 0 with the theoretical calculations (Fig. 3E). In our structure, there are several contributions to the linear polarization of polariton states. One is the transverse electric (TE)/transverse magnetic (TM) splitting, which is due to the intrinsic difference between the in- and out-of-plane effective refractive indices. Note that this energy splitting is bigger at higher **k** vectors. The second contribution comes from the in-plane optical anisotropy (crystal birefringence), which splits the modes at any **k** vectors along the two preferential axis. Lastly, in this material, there is also a polarization-dependent strong coupling of the two orientation of the exciton dipoles. Unlike TE/TM splitting, the other two contributions do not induce any polarization rotation. Figure 3 (B, C, F, and G) shows the DOP of modes well separated in energy, having an isotropic polarization in k_x_ and k_y_, due to the higher contribution of the birefringence compared to the TE/TM splitting. At the energy of about 1.574 eV, two different optical modes, respectively, coupled with one of the two excitons cross at high vector **k**, k_x_ ∼ ±6 μm^−1^. At this energy, the TE/TM effect begins to be observable, showing preferential coupling along the two dipole directions (see Fig. 3, D and H).

**Fig. 4.**
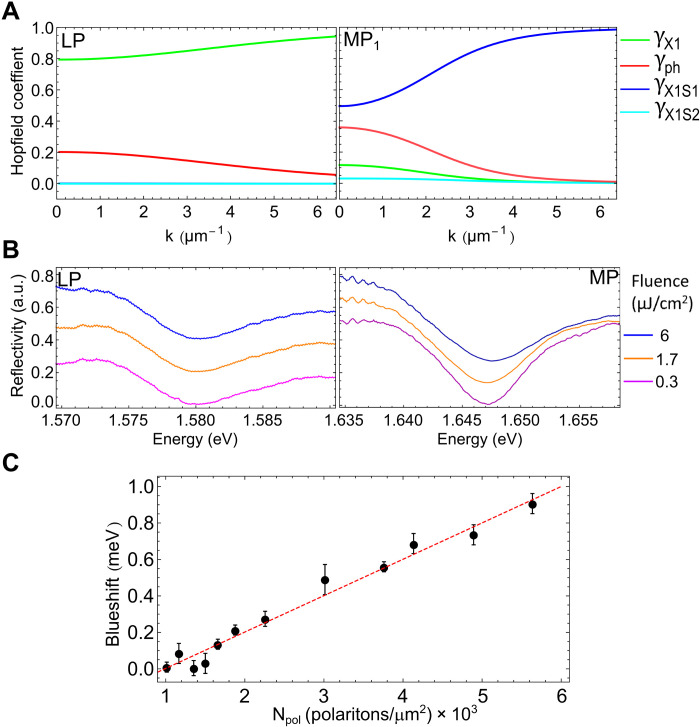
Hopfield coefficients and polariton nonlinearity. (**A**) Hopfield coefficients extracted for the lower (left) and first middle (right) polariton branch. (**B**) Reflection spectra obtained by resonantly exciting the lower and the middle polariton branches at **k** = 0 for different excitation pump powers. (**C**) Blueshift of the middle polariton by resonantly exciting the sample with a laser linearly polarized to the *b* axis; the dashed red line is the linear fit to the experimental data, resulting in a polariton-polariton interaction constant N_pol_(X1_S1_) = 0.2 μeV μm^2^.

The excited states, visible in Fig. 2B above 1.64 eV, also show strong coupling with same slab cavity mode. The values of the Rabi and the coupling strength for each exciton state are extracted by fitting our dispersions with a four coupled oscillator models (one mode, the ground exciton state, and two excited states). The result of this fitting is shown in fig. S7A with an extracted Rabi splitting of 84, 20, and 12 meV for the lower and the middle polariton states, respectively. By focusing on the Hopfield coefficients, which give the contribution of the excitons and the photon mode to the different polariton states, we can estimate the character of the polariton modes and how the polariton-polariton interaction should change in each of the three polariton branches. In Fig. 4A, which shows the Hopfield coefficients calculated for the lower and the first middle polaritonic bands, it can be seen that the lowest exciton-polariton mode (Fig. 4A, left) is practically decoupled from the excited excitons, and the state is predominantly having a ground-state character (with 80% of the fundamental exciton and only a 20% of photon contribution). Unexpectedly, the first middle polariton branch (Fig. 4A, right) is composed of three excitonic components, the fundamental and the two excited excitons. We can speculate then that such a state should lead to stronger polariton nonlinearity described with an interaction coefficient, *g*_*p*−*p*_, enhanced by the presence of higher-order excitons (*40*–*42*). For the excited states (called also Rydberg states), the radius of the different orbits scales as *n*^2^. Because the exciton-exciton interaction *g*_*X* − *X*_(*n*) depends linearly on the Bohr radius (*a*_B_), its intensity should increase quadratically: *g*_*X* − *X*_(*n*) = 6*E*_b_(*n*)*a*_B_^2^*n*^2^, where *E*_b_(*n*) is the binding energy of the *n*th state extracted by fitting the energy position of the different states in the absorption spectrum (fig. S8). Because the polariton-polariton interaction is given by the weighted sum of the different exciton contributions $g_{\;p-p}=\sum_{n}{\gamma_{X_{n}}}^{2}g_{X-X}(n)$, where γ*_X_n__* represents the normalized Hopfield coefficient for the *n*th state, it should be proportionally higher for the middle branch, which has higher content of the Rydberg excitons.

To experimentally observe this polariton-polariton interaction, we resonantly excite the ReS_2_ crystal with a fast, broadband, pulsed laser (∼50 fs) polarized along the *b* axis. We observe that whereas the reflection spectrum of the lower polariton state does not change by increasing the excitation power (Fig. 4B, left), possibly due to the limited nonlinearity of the ground-state exciton, the spectrum of the first middle polariton branch shows a small shift toward higher energies (Fig. 4B, right) (*43*, *44*). The energy blueshift of the reflection peak is plotted in Fig. 4C for the middle polariton state against the polariton density, resulting in a small but observable blueshift of about 1 meV. By fitting the experimental data (red shaded line in Fig. 4C), we are able to extract the polariton-polariton interaction constant *g*_pol_ (X1_S1_) = 0.2 μeV μm^2^. Although the second middle polariton should be characterized by a stronger polariton-polariton interaction, we do not observe any spectral shift with the pumping power. In general, we believe that because of the low quantum efficiency of this material (*45*), the real power density injected in resonant excitation is extremely low. This could be also the reason for such a small blueshift of the middle polariton branch and the lack of observable shift for the ground-state polariton.

In conclusion, we investigated the effect of the high refractive index and strong oscillator strength of the exciton transitions in ReS_2_ crystals and elucidated it. By hybridizing the ground-state excitons and the two first excited states, we are able to measure the effect of exciton-exciton interaction on the Rydberg states, showing a much stronger polariton-polariton interaction compared to the lower-energy state. Moreover, we have definitively demonstrated that this material has only two orthogonally polarized excitons, and we attributed the additional line in the reflectivity spectra to the longitudinal exciton feature. This work, beyond shedding light on the presence of different transition lines previously observed in ReS_2_ but mistakenly understood, provides new insights into this exciting material that could be successfully implemented in the realization of optical devices that exploit higher-order Rydberg states in solid-state materials.

## MATERIALS AND METHODS

ReS_2_ bulk crystals were grown by chemical vapor transport method using ICl_3_ as the transport agent (*9*). Because of the weak vDW bonding between the layers, micrometer-sized ReS_2_ flakes with desired thickness were obtained by mechanically exfoliating from bulk crystals and transferring them onto the final substrate by a dry transfer method (*46*) using commercial polydimethylsiloxane. The flakes were transferred on glass or DBR formed by eight pairs of SiO_2_/TiO_2_ with a stopband centered at λ_c_ = 785 nm and grown on glass substrate by electron-beam deposition (see the Supplementary Materials for details). By using a closed-cycle cryostat (attoDRY1000), the sample is cooled down to liquid helium temperature (*T* = 4 K) and excited by a white halogen lamp to measure the reflection spectra both in real and Fourier space. The polarized spectra along the *b* axis (V-polarized, ϕ = 0) and perpendicular to it (H-polarized, ϕ = 90) were obtained using a half wave plate and a polarizer placed in front of a spectrometer coupled to a charge-coupled device.

## Supplementary Materials

### This PDF file includes:

Supplementary Text Figs. S1 to S8 Tables S1 and S2 References

## References

[1] W. Choi, N. Choudhary, G. H. Han, J. Park, D. Akinwande, Y. H. Lee,Recent development of two-dimensional transition metal dichalcogenides and their applications. Mater. Today 20,116–130 (2017).

[2] A. T. Hanbicki, M. Currie, G. Kioseoglou, A. L. Friedman, B. T. Jonker,Measurement of high exciton binding energy in the monolayer transition-metal dichalcogenides WS_2_ and WSe_2_. Solid State Commun. 203,16–20 (2015).

[3] A. Chernikov, T. C. Berkelbach, H. M. Hill, A. Rigosi, Y. Li, O. B. Aslan, D. R. Reichman, M. S. Hybertsen, T. F. Heinz,Exciton binding energy and nonhydrogenic Rydberg series in monolayer WS_2_. Phys. Rev. Lett. 113,076802 (2014). 25170725 10.1103/PhysRevLett.113.076802

[4] F. Cadiz, E. Courtade, C. Robert, G. Wang, Y. Shen, H. Cai, T. Taniguchi, K. Watanabe, H. Carrere, D. Lagarde,Excitonic linewidth approaching the homogeneous limit inMoS2-Based van der Waals heterostructures. Phys. Rev. X 7,021026 (2017).

[5] X. Xu, W. Yao, D. Xiao, T. F. Heinz,Spin and pseudospins in layered transition metal dichalcogenides. Nat. Phys. 10,343–350 (2014).

[6] F. Koppens, T. Mueller, P. Avouris, A. Ferrari, M. Vitiello, M. Polini,Photodetectors based on graphene, other two-dimensional materials and hybrid systems. Nat. Nanotechnol. 9,780–793 (2014). 25286273 10.1038/nnano.2014.215

[7] K. F. Mak, J. Shan,Photonics and optoelectronics of 2D semiconductor transition metal dichalcogenides. Nat. Photonics 10,216–226 (2016).

[8] Y. Sun, D. Wang, Z. Shuai,Indirect-to-direct band gap crossover in few-layer transition metal dichalcogenides: A theoretical prediction. J. Phys. Chem. C 120,21866–21870 (2016).

[9] C.-H. Ho, Z.-Z. Liu,Complete-series excitonic dipole emissions in few layer ReS_2_ and ReSe_2_ observed by polarized photoluminescence spectroscopy. Nano Energy 56,641–650 (2019).

[10] C. H. Ho, Y. S. Huang, K.-K. Tiong,In-plane anisotropy of the optical and electrical properties of ReS_2_ and ReSe_2_ layered crystals. J. Alloys Compd. 317,222–226 (2001).

[11] Y. Y. Wang, J. D. Zhou, J. Jiang, T. T. Yin, Z. X. Yin, Z. Liu, Z. X. Shen,In-plane optical anisotropy in ReS_2_ flakes determined by angle-resolved polarized optical contrast spectroscopy. Nanoscale 11,20199–20205 (2019). 31617546 10.1039/c9nr07502j

[12] A. A. Shubnic, R. G. Polozkov, I. A. Shelykh, I. V. Iorsh,High refractive index and extreme biaxial optical anisotropy of rhenium diselenide for applications in all-dielectric nanophotonics. Nanophotonics 9,4737–4742 (2020).

[13] E. Zhang, Y. Jin, X. Yuan, W. Wang, C. Zhang, L. Tang, S. Liu, P. Zhou, W. Hu, F. Xiu,ReS_2_-based field-effect transistors and photodetectors. Adv. Funct. Mater. 25,4076–4082 (2015).

[14] E. Liu, Y. Fu, Y. Wang, Y. Feng, H. Liu, X. Wan, W. Zhou, B. Wang, L. Shao, C.-H. Ho, Y.-S. Huang, Z. Cao, L. Wang, A. Li, J. Zeng, F. Song, X. Wang, Y. Shi, H. Yuan, H. Y. Hwang, Y. Cui, F. Miao, D. Xing,Integrated digital inverters based on two-dimensional anisotropic ReS_2_ field-effect transistors. Nat. Commun. 6,1–7 (2015). 10.1038/ncomms7991 PMC4432591 25947630

[15] J. Shim, A. Oh, D.-H. Kang, S. Oh, S. K. Jang, J. Jeon, M. H. Jeon, M. Kim, C. Choi, J. Lee, S. Lee, G. Y. Yeom, Y. J. Song, J.-H. Park,High-performance 2D rhenium disulfide (ReS_2_) transistors and photodetectors by oxygen plasma treatment. Adv. Mater. 28,6985–6992 (2016). 27206245 10.1002/adma.201601002

[16] Q. Zhang, W. Wang, J. Zhang, X. Zhu, Q. Zhang, Y. Zhang, Z. Ren, S. Song, J. Wang, Z. Ying, R. Wang, X. Qiu, T. Peng, L. Fu,Highly efficient photocatalytic hydrogen evolution by ReS_2_ via a two-electron catalytic reaction. Adv. Mater. 30,e1707123 (2018). 29687485 10.1002/adma.201707123

[17] Q. Zhang, L. Fu,Novel insights and perspectives into weakly coupled ReS_2_ toward emerging applications. Chem 5,505–525 (2019).

[18] S. Tongay, H. Sahin, C. Ko, A. Luce, W. Fan, K. Liu, J. Zhou, Y.-S. Huang, C.-H. Ho, J. Yan, D. Frank Ogletree, S. Aloni, J. Ji, S. Li, J. Li, F. M. Peeters, J. Wu,Monolayer behaviour in bulk ReS_2_ due to electronic and vibrational decoupling. Nat. Commun. 5,1–6 (2014). 10.1038/ncomms4252 24500082

[19] J. B. Khurgin,Expanding the photonic palette: Exploring high index materials. ACS Photonics 9,743–751 (2022).

[20] K. Rechcińska, M. Król, R. Mazur, P. Morawiak, R. Mirek, K. Łempicka, W. Bardyszewski, M. Matuszewski, P. Kula, W. Piecek, P. G. Lagoudakis, B. Piętka, J. Szczytko,Engineering spin-orbit synthetic Hamiltonians in liquid-crystal optical cavities. Science 366,727–730 (2019). 31699934 10.1126/science.aay4182

[21] E. Altman, L. M. Sieberer, L. Chen, S. Diehl, J. Toner,Two-dimensional superfluidity of exciton polaritons requires strong anisotropy. Phys. Rev. X 5,011017 (2015).

[22] R. Gogna, L. Zhang, H. Deng,Self-hybridized, polarized polaritons in ReS_2_ crystals. ACS Photonics 7,3328–3332 (2020).

[23] D. Chakrabarty, A. Dhara, K. Ghosh, A. K. Pattanayak, S. Mukherjee, A. R. Chaudhuri, S. Dhara,Interfacial anisotropic exciton-polariton manifolds in ReS_2_. Optica 8,1488–1494 (2021).

[24] A. Dhara, D. Chakrabarty, P. Das, A. K. Pattanayak, S. Paul, S. Mukherjee, S. Dhara,Additional excitonic features and momentum-dark states in ReS_2_. Phys. Rev. B 102,161404 (2020).

[25] A. Arora, J. Noky, M. Drüppel, B. Jariwala, T. Deilmann, R. Schneider, R. Schmidt, O. Del Pozo-Zamudio, T. Stiehm, A. Bhattacharya,Highly anisotropic in-plane excitons in atomically thin and bulklike 1 T′-ReSe. Nano Lett. 17,3202–3207 (2017). 28414459 10.1021/acs.nanolett.7b00765

[26] C. H. Liang, Y. H. Chan, K.-K. Tiong, Y. S. Huang, Y. M. Chen, D. O. Dumcenco, C. H. Ho,Optical anisotropy of Au-doped ReS_2_ crystals. J. Alloys Compd. 480,94–96 (2009).

[27] D. A. Chenet, O. B. Aslan, P. Y. Huang, C. Fan, A. M. Van Der Zande, T. F. Heinz, J. C. Hone,In-plane anisotropy in mono- and few-layer ReS_2_ probed by Raman spectroscopy and scanning transmission electron microscopy. Nano Lett. 15,5667–5672 (2015). 26280493 10.1021/acs.nanolett.5b00910

[28] O. B. Aslan, D. A. Chenet, A. M. Van Der Zande, J. C. Hone, T. F. Heinz,Linearly polarized excitons in single- and few-layer ReS_2_ crystals. Acs Photonics 3,96–101 (2016).

[29] S. Sim, D. Lee, M. Noh, S. Cha, C. H. Soh, J. H. Sung, M.-H. Jo, H. Choi,Selectively tunable optical Stark effect of anisotropic excitons in atomically thin ReS_2_. Nat. Commun. 7,1–6 (2016). 10.1038/ncomms13569 PMC5120211 27857053

[30] J. Jadczak, J. Kutrowska-Girzycka, T. Smoleński, P. Kossacki, Y. Huang, L. Bryja,Exciton binding energy and hydrogenic Rydberg series in layered ReS_2_. Sci. Rep. 9,1–9 (2019). 30733485 10.1038/s41598-018-37655-8 PMC6367321

[31] J. Wang, Y. J. Zhou, D. Xiang, S. J. Ng, K. Watanabe, T. Taniguchi, G. Eda,Polarized light-emitting diodes based on anisotropic excitons in few-layer ReS_2_. Adv. Mater. 32,2001890 (2020). 10.1002/adma.202001890 32608083

[32] H. Deng, H. Haug, Y. Yamamoto,Exciton-polariton bose-einstein condensation. Rev. Mod. Phys. 82,1489–1537 (2010).

[33] J. J. Hopfield,Theory of the contribution of excitons to the complex dielectric constant of crystals. Phys. Rev. 112,1555–1567 (1958).

[34] L. C. Andreani, F. Bassani, A. Quattropani,Longitudinal-transverse splitting in Wannier excitons and polariton states. Il Nuovo Cimento D 10,1473–1486 (1988).

[35] M. M. Denisov, V. P. Makarov,Longitudinal and transverse excitons in semiconductors. Phys. Status Solidi B 56,9–59 (1973).

[36] A. Canales, D. G. Baranov, T. J. Antosiewicz, T. Shegai,Abundance of cavity-free polaritonic states in resonant materials and nanostructures. J. Chem. Phys. 154,024701 (2021). 33445887 10.1063/5.0033352

[37] V. Liu, S. Fan,S4: A free electromagnetic solver for layered periodic structures. Comput. Phys. Commun. 183,2233–2244 (2012).

[38] Q. Cui, J. He, M. Z. Bellus, M. Mirzokarimov, T. Hofmann, H. Chiu, M. Antonik, D. He, Y. Wang, H. Zhao,Transient absorption measurements on anisotropic monolayer ReS_2_. Small 11,5565–5571 (2015). 26317682 10.1002/smll.201501668

[39] T. Wen, J. Li, Q. Deng, C. Jiao, M. Zhang, S. Wu, L. Lin, W. Huang, J. Xia, Z. Wang,Analyzing anisotropy in 2D rhenium disulfide using dichromatic polarized reflectance. Small 18,2108028 (2022). 10.1002/smll.202108028 35315231

[40] J. Gu, V. Walther, L. Waldecker, D. Rhodes, A. Raja, J. C. Hone, T. F. Heinz, S. Kéna-Cohen, T. Pohl, V. M. Menon,Enhanced nonlinear interaction of polaritons via excitonic Rydberg states in monolayer WSe_2_. Nat. Commun. 12,1–7 (2021). 33859179 10.1038/s41467-021-22537-x PMC8050076

[41] T. Yagafarov, D. Sannikov, A. Zasedatelev, K. Georgiou, A. Baranikov, O. Kyriienko, I. Shelykh, L. Gai, Z. Shen, D. Lidzey, P. Lagoudakis,Mechanisms of blueshifts in organic polariton condensates. Commun. Phys. 3,1–10 (2020).

[42] K. Orfanakis, S. K. Rajendran, V. Walther, T. Volz, T. Pohl, H. Ohadi,Rydberg exciton–Polaritons in a Cu_2_O microcavity. Nat. Mater. 21,1–6 (2022). 35422507 10.1038/s41563-022-01230-4

[43] V. Walther, R. Johne, T. Pohl,Giant optical nonlinearities from Rydberg excitons in semiconductor microcavities. Nat. Commun. 9,1–6 (2018). 29615612 10.1038/s41467-018-03742-7 PMC5883042

[44] X. Liu, T. Galfsky, Z. Sun, F. Xia, E. Lin, Y.-H. Lee, S. Kéna-Cohen, V. M. Menon,Strong light–Matter coupling in two-dimensional atomic crystals. Nat. Photonics 9,30–34 (2015).

[45] N. B. Mohamed, K. Shinokita, X. Wang, H. E. Lim, D. Tan, Y. Miyauchi, K. Matsuda,Photoluminescence quantum yields for atomically thin-layered ReS_2_: Identification of indirect-bandgap semiconductors. Appl. Phys. Lett. 113,121112 (2018).

[46] Y. Chen, X.-L. Gong, J.-G. Gai,Progress and challenges in transfer of large-area graphene films. Adv. Sci. 3,1500343 (2016). 10.1002/advs.201500343 PMC5067701 27812479

[47] C. F. Klingshirn, *Semiconductor Optics* (Springer, 2012).

