# Supplementary material for: Rydberg polaritons in ReS2 crystals
Source: Sci Adv. 2022 Nov 23;8(47):eadd8857. doi: 10.1126/sciadv.add8857 (PMC9683695; doi:10.1126/sciadv.add8857)
Supplement: Supplementary file 1 — Supplementary Text Figs. S1 to S8 Tables S1 and S2 References [file sciadv.add8857_sm.pdf]

Science Advances
AAAS

Supplementary Materials for

Rydberg polaritons in ReS_2 crystals

Annalisa Coriolano *et al.*

Corresponding author: Milena De Giorgi, milena.degiorgi@nanotec.cnr.it

Sci. Adv. **8**, eadd8857 (2022)
DOI: 10.1126/sciadv.add8857

This PDF file includes:

Supplementary Text
Figs. S1 to S8
Tables S1 and S2
References

ReS_2 crystal growth and sample preparation

Layered single crystals of ReS_2 were are grown by chemical vapor transport (CVT) using ICl_3 as the transport agent, as reported in a previous work (*9*). Thanks to weak Van der Waals force between the layers, few-layers of ReS_2 are obtained by mechanical exfoliation using commercial PDMS (Gel Pak) and transferred on glass or DBR substrates. Due to the in-plane mechanical anisotropy along the Re chains, the crystal b-axis is identified as a longer crystal edge, as shown in Fig.2A of the main text, obtained after the mechanical exfoliation. To improve the crystal adhesion on the substrate and optimize the flakes quality, we pre-treated the surface of the DBR substrate with 4-(2-aminethyl) benzoic acid in order to create a self-assembled monolayer (SAM): the substrate was immersed in a 0.13 %wt solution of 4-(2-aminethyl) benzoic acid hydrochloride in ethanol for 24 hours at room temperature and then rinsed with ethanol to remove excess molecules.

Distributed Bragg Reflector

The DBR is formed by eight pairs of SiO_2/TiO_2 layers (with thicknesses of 136 nm/93 nm, respectively), deposited by electron-beam deposition (Temescal Supersource) in vacuum, keeping the chamber at $10^{-5} \div 10^{-6}$ mbar throughout the process, at room temperature (deposition rates: 1 Å/s for SiO_2, 0.5 Å/s for TiO_2). The DBR is deposited on top of a 170 µm glass substrate and the resulting stopband is centered at 785 nm.

Optical measurements

All optical measurements are performed in reflection configuration in a helium cryostat at cryogenic temperature (T=4K) using a halogen white lamp. The photoluminescence is recorded in reflection configuration with a continuous-wave laser, centered at λ = 488 nm. The spectra are collected with an 50x objective, with a numerical aperture NA= 0.82. The polarizer and the half-wave plate (HWP) on the detection path allow for the measurement of the polarization-resolved spectra, both in real space and in back focal space.

Theoretical Simulations

Our structure has been simulated by using the semi-analytical Rigorous Coupled-Wave Analysis (RCWA) method implemented by S4 package (*37*). The exciton resonances, E_{X1} and E_{X2}, have been modeled using a Lorentz oscillator, with a dielectric function given by:

$$\varepsilon_{1,2} = \varepsilon_{B_{1,2}} + \frac{f_{1,2}}{E_{x_{1,2}}^2 - E^2 - i\,E\,\Gamma_{1,2}}$$

where:

ϵ_B is the background permittivity

E_X is the exciton resonance energy

Γ is the exciton linewidth

f is the oscillator strength

After the measurement of the ReS_2 crystals thickness by AFM, we simulate the experimental reflectance spectra by using the following parameters, summarized in Tab.S1.

	ϵ_B	***f [eV^2]***	***Γ [eV]***
Polarization // to b-axis (V)	17	$f_{x1} = 0.3$	$\Gamma_{x1} = 0.003$
		$f_{S1} = 0.03$	$\Gamma_{S1} = 0.0025$
		$f_{S2} = 0.022$	$\Gamma_{S2} = 0.0025$
		$f_{S3} = 0.01$	$\Gamma_{S3} = 0.0015$
		$f_{S4} = 0.01$	$\Gamma_{S4} = 0.0015$
Polarization ⊥ to b-axis (H)	13.5	$f_{x2} = 0.25$	$\Gamma_{x2} = 0.003$
		$f_{S1} = 0.018$	$\Gamma_{S1} = 0.002$
		$f_{S2} = 0.001$	$\Gamma_{S2} = 0.002$
		$f_{S3} = 0.001$	$\Gamma_{S3} = 0.001$

Table S1. Background permittivity, oscillator strength and full with at half maximum for the different states. Values of the parameters utilized in the RCWA simulations.

Theoretical Simulations of ReS_2 crystal in vacuum

Fig. S1 shows the real part of the permittivity by fixing the value of background permittivity, ε_B, for different oscillator strength (*f*). We consider a 60 nm – thick ReS_2 crystal immersed in vacuum and simulate the energy dispersion of the reflectivity for different values of oscillator strength. The energy splitting $\Delta E_{L/T}$ increases for higher coupling strength between the exciton and photons.

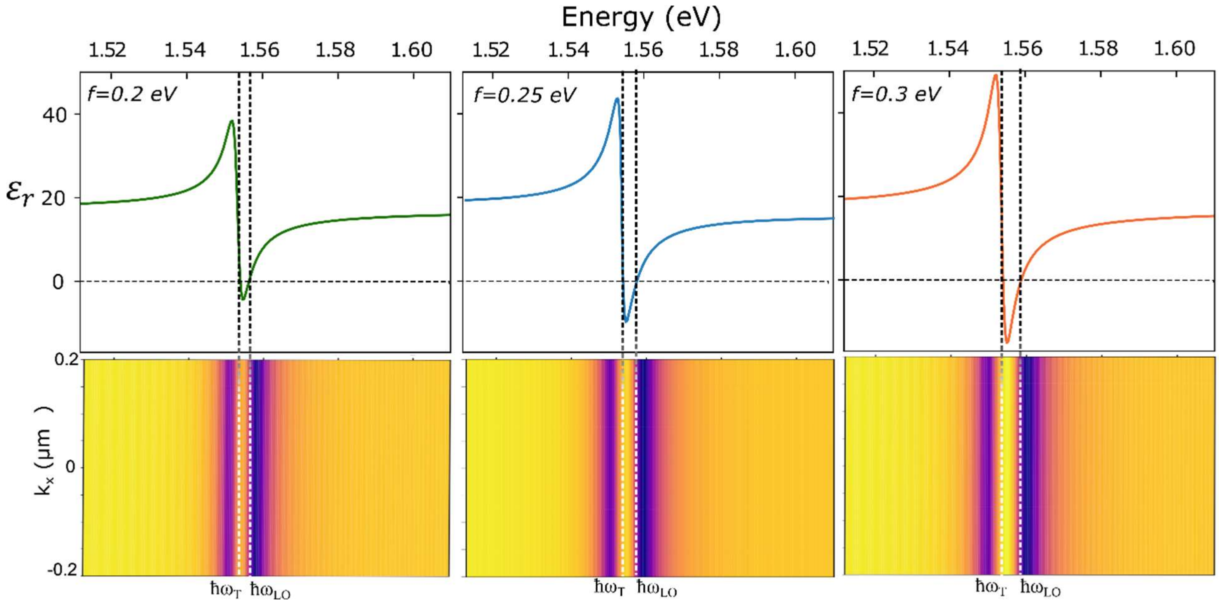

Fig. S1. Exciton energy splitting dependence on oscillator strength. Top panels) Real part of the permittivity for different values of oscillator strength. The correspondent energy dispersions of the reflectivity for different values of f are reported in the bottom panels.

Theoretical simulation of ReS_2 reflectivity in function of the thickness

The reflectivity spectra for different thickness of ReS_2 crystals on glass substrate are reported in Fig.S2. Additional resonances around the main exciton are clearly distinguishable for thick crystals (≥ 50 nm), whereas for the thinner one only the main exciton transitions are distinctly observable.

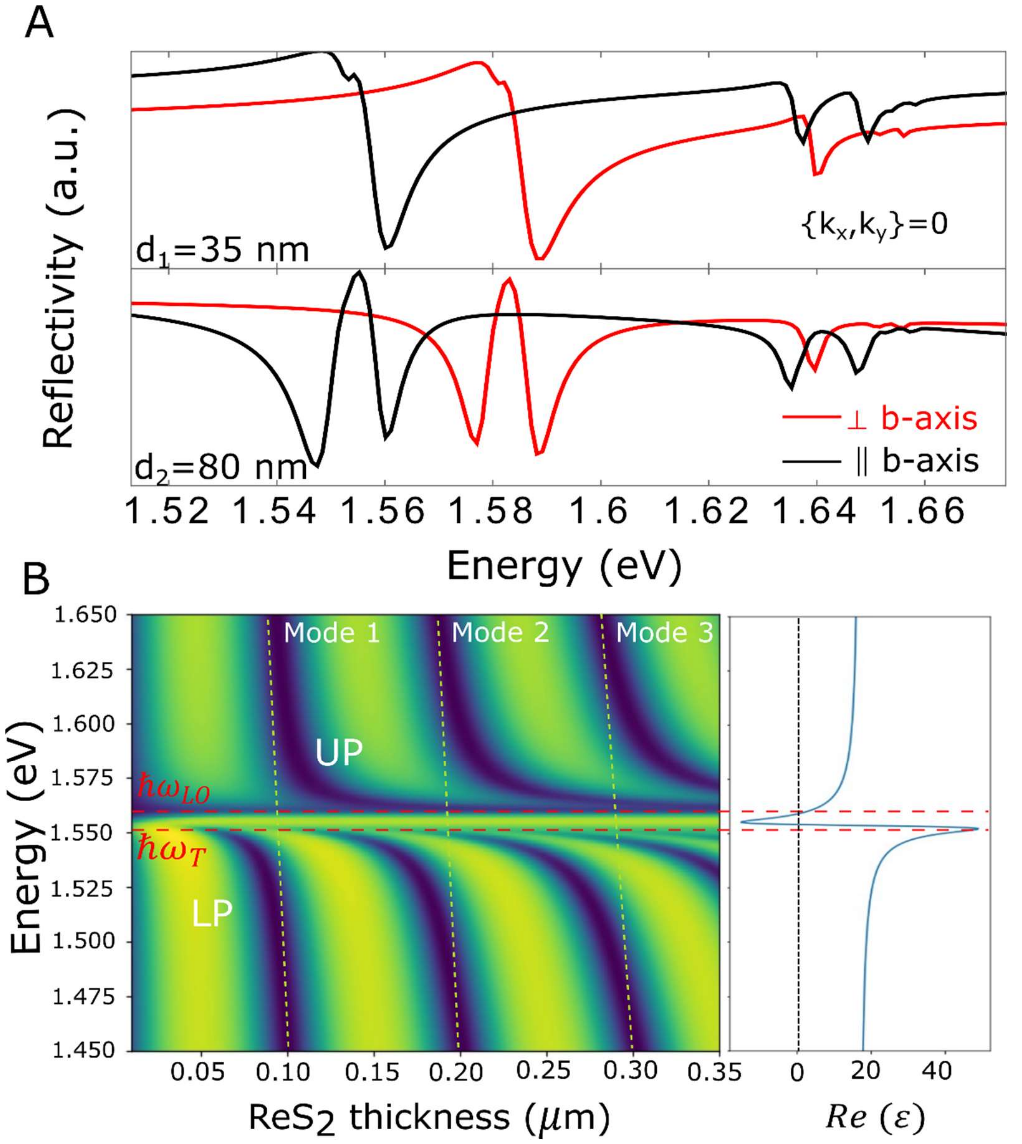

Fig. S2. **Reflectivity spectra for different ReS_2 crystal thicknesses.** (**A**) Theoretical reflectivity spectra of crystals with thickness of 35 nm (Top panel) and 80 nm (Bottom panel). (**B**) (Left panel) Theoretical map of the reflectivity versus crystal thickness for vertical polarization assuming only an exciton resonance at 1.554 eV and unpolarized light incident perpendicular to the surface crystal. The dashed red lines are the longitudinal and transversal modes for the ReS_2 crystals which couple to the exciton resulting in a new hybrid states, lower (LP) and upper (UP) polariton bands tending asymptotically at the longitudinal (L) and transversal (T) modes. (Right panel) Real part of the theoretical permittivity in the energy range of the reflectivity map.

Atomic Force Microscopy (AFM)

AFM measurements are performed using a Park Scanning Probe Microscope (PSIA), with a high resonant frequency non-contact cantilever. The image acquisition is performed in air at room temperature.

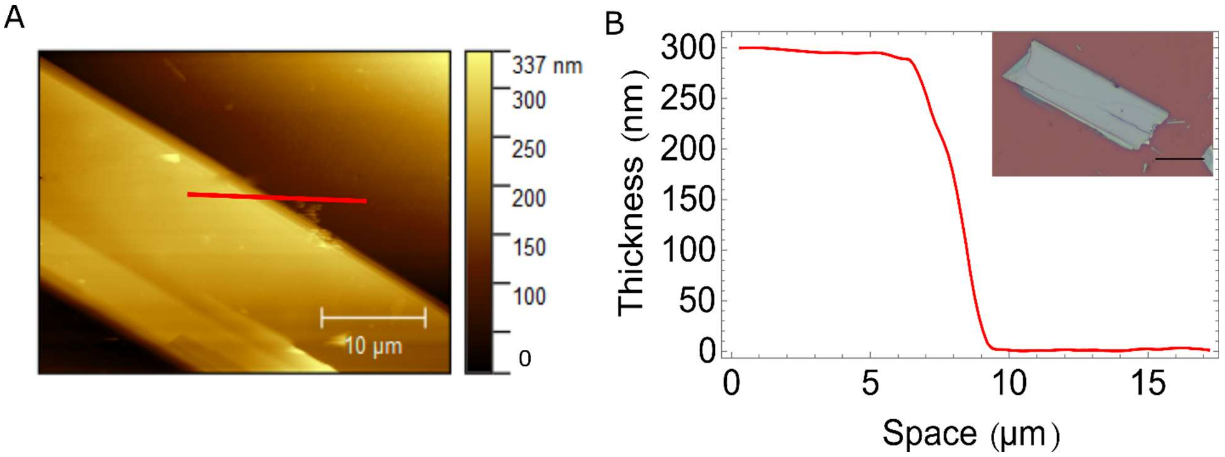

Fig. S3. Topographic analysis. (**A**) AFM topography image of 310 nm – thick ReS_2 crystal on DBR. (**B**) The corresponding height profile measured along the red line. (Inset) Optical image of the crystal. The scalebar is 20 μm.

Theoretical Simulations of the ReS_2 structure on top of DBR

We simulate the reflectance spectra at $\{k_x, k_y\} = 0$ for different thickness of the ReS_2 crystals on DBR, showing a different coupling effect for the two polarizations, in particular on the excited states.

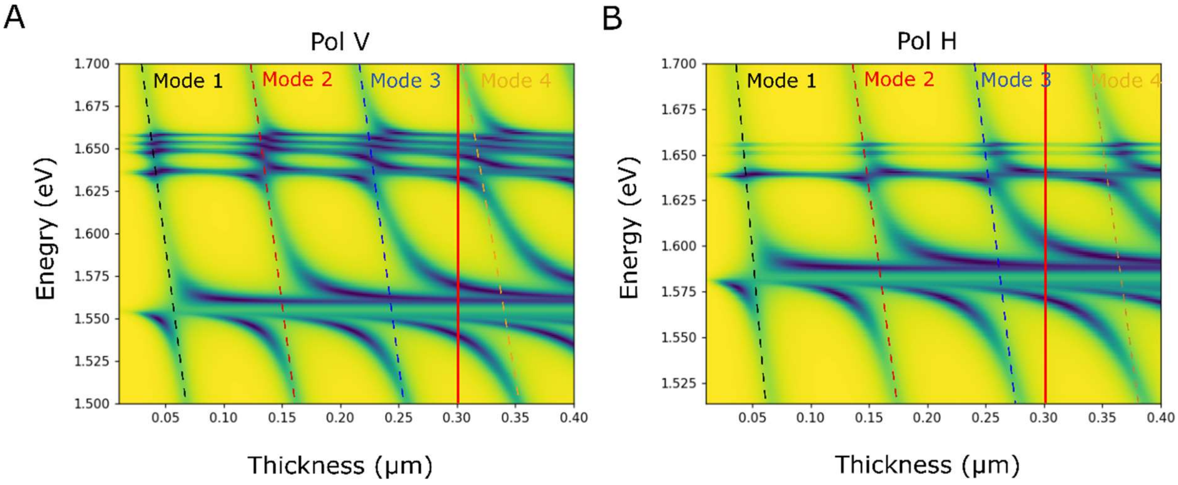

Fig. S4. Cavity effect on the reflectivity for different crystal thicknesses. Theoretical map of the reflectivity versus crystal thickness for the vertical (**A**) and horizontal polarization (**B**) assuming unpolarized light incident perpendicular to the surface crystal. The vertical red lines mark the energy

dispersions for both polarizations for a crystal 310 nm thick, in agreement with the experimental data reported in Fig.2 **A-B**.

Tuning of ReS_2 crystal thickness

By changing the ReS_2 crystal thickness, we tune the coupling of the exciton and the excited states with different photonic modes: in a 60 nm-thick crystal, both excitons couple with the photonic mode 1, leading to the formation of the lower polariton states and the middle ones, while the excited states are uncoupled for both the polarizations (see Fig.S5).

For thicker sample (d = 140 nm), we can measure the coupling of the excitons with both the photonic Mode "2" and Mode "1", resulting in a more structured dispersion spectrum (see Fig.S6). Analyzing the V polarization spectra for different thickness, we conclude that the different detuning of the photonic modes with respect to the exciton E_{X1} results in a different exciton component of the lower polariton branches, which becomes more excitonic from d= 310 nm to d = 60 nm (see Fig.S7).

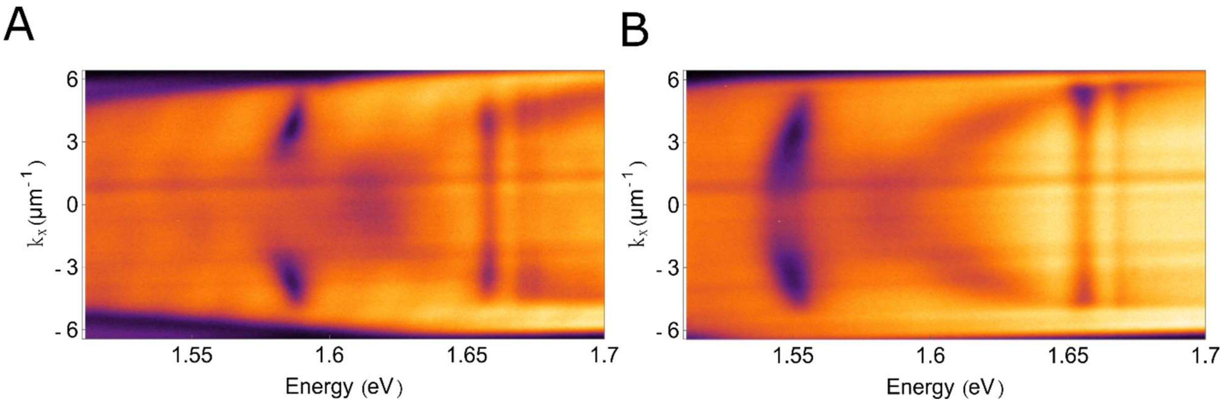

Fig. S5. Polarized reflection spectra for a crystal of 60 nm. Energy vs kx in-plane momentum of reflection spectra polarized perpendicular to the b – axis (**A**) and parallel to the b – axis (**B**) for a 60 nm – thick ReS_2 crystal on a DBR.

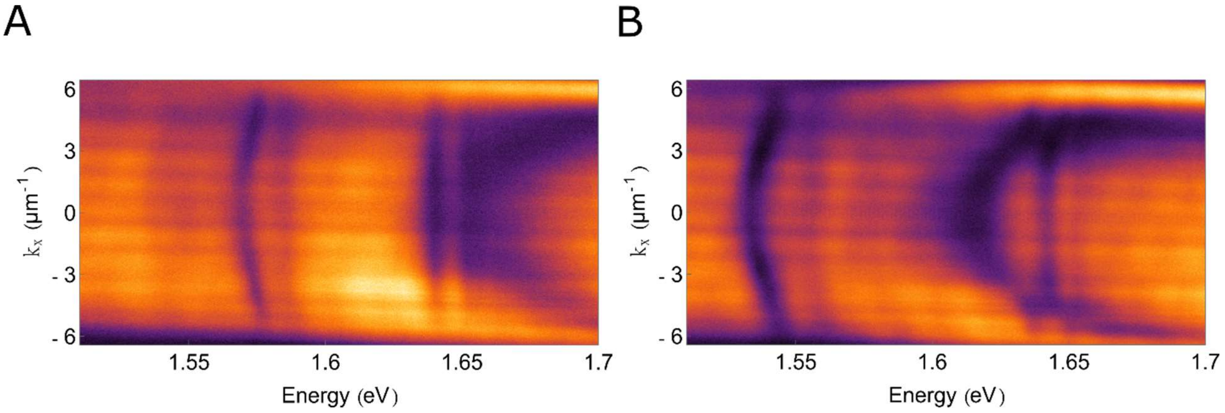

Fig. S6. Polarized reflection spectra for a crystal of 140 nm. Energy vs kx in-plane momentum of reflection spectra polarized perpendicular to the b – axis (**A**) and parallel to the b – axis (**B**) for a 140 nm – thick ReS_2 crystal on a DBR.

To extract the Rabi splitting values from reflection spectra reported in Fig.2B-C of the main text, we diagonalize a four coupled oscillator system matrix, described as follow:

$$\begin{pmatrix} E_{X1}+\frac{\hbar^2k^2}{2m_{x1}} & 0 & 0 & \Omega_{X1} \\ 0 & E_{X1S1}+\frac{\hbar^2k^2}{2m_{X1S1}} & 0 & \Omega_{X1S1} \\ 0 & 0 & E_{X1S2}+\frac{\hbar^2k^2}{2m_{X1S2}} & \Omega_{X1S2} \\ \Omega_{X1} & \Omega_{X1S1} & \Omega_{X1S2} & E_C+\frac{\hbar^2k^2}{2m_C} \end{pmatrix} \quad (1)$$

in which $E_{X1} = 1.554\ eV$, $E_{X1S1} = 1.636\ eV$ and $E_{X1S2} = 1.648\ eV$ are the experimental energy resonances of the exciton and two excited states, respectively, while $E_C = 1.617\ eV$ is the photon energy.

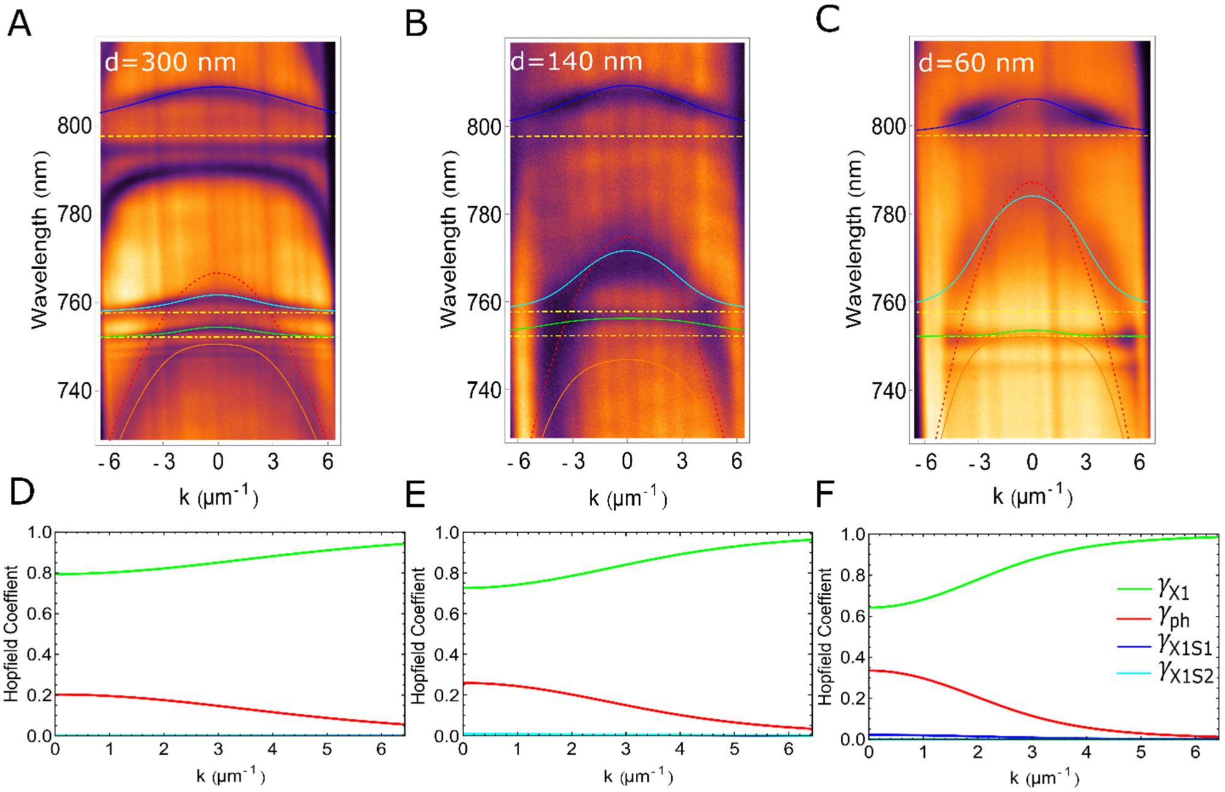

Fig. S7. **Strong coupling and Hopfield coefficients for different crystal thicknesses.** (**A-C**) Different polariton branches for ReS_2 crystals with thickness of d = 310 nm (**A**), d = 140 nm (**B**), and d = 60 nm (**C**), obtained by assuming the coupling between three exciton states (shown with the yellow dashed lines, E_{X1}, E_{X1S1} and E_{X1S2}) with only one photonic mode (red dotted lines), which results in a lower polariton branch (blue lines), two middle polariton branches (cyan and green lines) and an upper polariton branch (orange line). (**D-F**) The Hopfield coefficient for the lower polariton branch for the considered ReS_2 thickness.

Fig.S7A-C show the fitting of different reflection spectra as function of the in-plane momentum, decreasing the ReS_2 crystal thickness. We extract the Rabi splitting value of 84 meV, 74 meV and 44 meV for 310 nm, 140 nm and 60 nm flakes thickness, respectively.
In order to calculate the exciton and photon fraction of the polariton branches for different thickness, we evaluate the Hopfield coefficients (Fig.S7D-F) from the eigenvectors of the matrix (1). In this way, we consider not only the contribution of the exciton E_{X1} and the photonic mode to the exciton fraction, but also the effect of the two excited states E_{X1S1} and E_{X1S2}. Note that in Fig.S7A, the exciton energy (dashed yellow line around 798 nm) is between the longitudinal (L) and transversal (T) modes generated in the ReS_2 crystals which are clearly evident in the experimental dispersion spectra for the thickness of d=310nm in Fig.2B.

Rydberg States
The reflectance spectra of thicker ReS_2 crystals show peaks at higher energy, in addition to the transitions of the fundamental excitons. These states are excited states of both the excitons and their energies follow the usual hydrogenic Rydberg series of energy levels of 3D excitonic states ($En = Ry^*/n^2$).

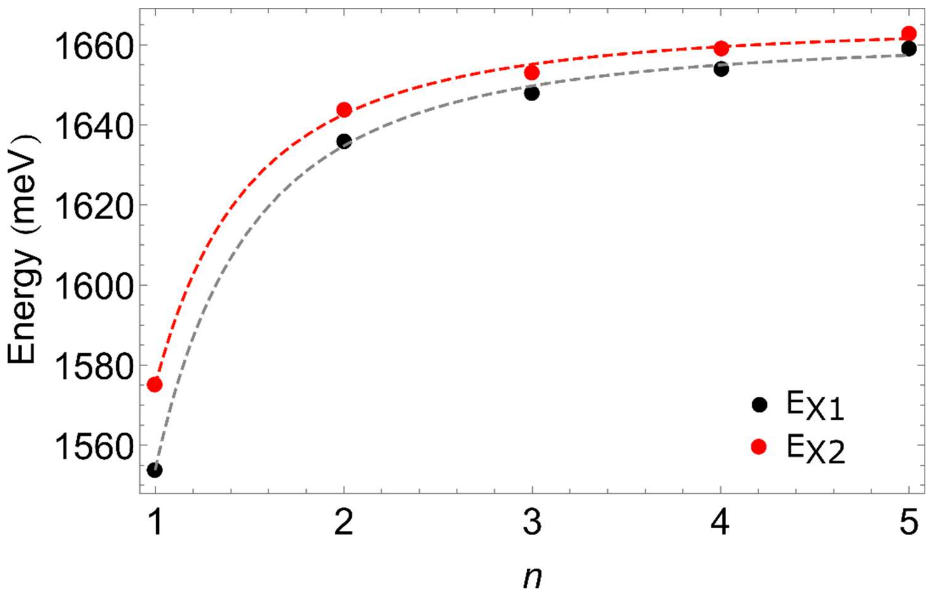

Fig. S8. **Rydberg series corresponding to the two ReS_2 excitons.** Experimental data (dots) and theoretical prediction of hydrogenic Rydberg series (dashed lines) of energy exciton states as a function of the quantum number n, for the E_{X1} (black) and E_{X2} (red) excitons.

The formula used to extract the binding energy of the excited states describing the three dimensional Wannier excitons in inorganic semiconductor (*47*) is:

$$E_b^{(n)} = E_g - Ry^*/n^2$$

where $E_b^{(n)}$ is the binding energy at the nth excitonic state, E_g is the energy gap of the ReS_2, Ry^* is the effective Rydberg constant and n is the number of exciton state. The fitting parameters

extracted for the two Rydberg series associated with the two excitons (for the two polarizations) are reported in Tab.S2.

	E_g (meV)	R_y (meV)	$E_b^{(Ex)}$ (meV)	$E_b^{(1)}$ (meV)	$E_b^{(2)}$ (meV)	$E_b^{(3)}$ (meV)	$E_b^{(4)}$ (meV)
Polarization // to b-axis (V)	1661	107	107.58	26.89	11.9	6.72	4.3
Polarization ⊥ to b-axis (H)	1665	90	90.03	22.5	10	5.63	3.6

Table S2. Rydberg Parameters. Fitting parameters extracted for the excited state of the two excitons E_{x1} and E_{x2}.

REFERENCES AND NOTES

1. W. Choi, N. Choudhary, G. H. Han, J. Park, D. Akinwande, Y. H. Lee, Recent development of two-dimensional transition metal dichalcogenides and their applications. *Mater. Today* **20**, 116–130 (2017).

2. A. T. Hanbicki, M. Currie, G. Kioseoglou, A. L. Friedman, B. T. Jonker, Measurement of high exciton binding energy in the monolayer transition-metal dichalcogenides WS_2 and WSe_2. *Solid State Commun.* **203**, 16–20 (2015).

3. A. Chernikov, T. C. Berkelbach, H. M. Hill, A. Rigosi, Y. Li, O. B. Aslan, D. R. Reichman, M. S. Hybertsen, T. F. Heinz, Exciton binding energy and nonhydrogenic Rydberg series in monolayer WS_2. *Phys. Rev. Lett.* **113**, 076802 (2014).

4. F. Cadiz, E. Courtade, C. Robert, G. Wang, Y. Shen, H. Cai, T. Taniguchi, K. Watanabe, H. Carrere, D. Lagarde, Excitonic linewidth approaching the homogeneous limit inMoS2-Based van der Waals heterostructures. *Phys. Rev. X* **7**, 021026 (2017).

5. X. Xu, W. Yao, D. Xiao, T. F. Heinz, Spin and pseudospins in layered transition metal dichalcogenides. *Nat. Phys.* **10**, 343–350 (2014).

6. F. Koppens, T. Mueller, P. Avouris, A. Ferrari, M. Vitiello, M. Polini, Photodetectors based on graphene, other two-dimensional materials and hybrid systems. *Nat. Nanotechnol.* **9**, 780–793 (2014).

7. K. F. Mak, J. Shan, Photonics and optoelectronics of 2D semiconductor transition metal dichalcogenides. *Nat. Photonics* **10**, 216–226 (2016).

8. Y. Sun, D. Wang, Z. Shuai, Indirect-to-direct band gap crossover in few-layer transition metal dichalcogenides: A theoretical prediction. *J. Phys. Chem. C* **120**, 21866–21870 (2016).

9. C.-H. Ho, Z.-Z. Liu, Complete-series excitonic dipole emissions in few layer ReS_2 and $ReSe_2$ observed by polarized photoluminescence spectroscopy. *Nano Energy* **56**, 641–650 (2019).

10. C. H. Ho, Y. S. Huang, K.-K. Tiong, In-plane anisotropy of the optical and electrical properties of ReS_2 and $ReSe_2$ layered crystals. *J. Alloys Compd.* **317**, 222–226 (2001).

11. Y. Y. Wang, J. D. Zhou, J. Jiang, T. T. Yin, Z. X. Yin, Z. Liu, Z. X. Shen, In-plane optical anisotropy in ReS_2 flakes determined by angle-resolved polarized optical contrast spectroscopy. *Nanoscale* **11**, 20199–20205 (2019).

12. A. A. Shubnic, R. G. Polozkov, I. A. Shelykh, I. V. Iorsh, High refractive index and extreme biaxial optical anisotropy of rhenium diselenide for applications in all-dielectric nanophotonics. *Nanophotonics* **9**, 4737–4742 (2020).

13. E. Zhang, Y. Jin, X. Yuan, W. Wang, C. Zhang, L. Tang, S. Liu, P. Zhou, W. Hu, F. Xiu, ReS_2-based field-effect transistors and photodetectors. *Adv. Funct. Mater.* **25**, 4076–4082 (2015).

14. E. Liu, Y. Fu, Y. Wang, Y. Feng, H. Liu, X. Wan, W. Zhou, B. Wang, L. Shao, C.-H. Ho, Y.-S. Huang, Z. Cao, L. Wang, A. Li, J. Zeng, F. Song, X. Wang, Y. Shi, H. Yuan, H. Y. Hwang, Y. Cui, F. Miao, D. Xing, Integrated digital inverters based on two-dimensional anisotropic ReS_2 field-effect transistors. *Nat. Commun.* **6**, 1–7 (2015).

15. J. Shim, A. Oh, D.-H. Kang, S. Oh, S. K. Jang, J. Jeon, M. H. Jeon, M. Kim, C. Choi, J. Lee, S. Lee, G. Y. Yeom, Y. J. Song, J.-H. Park, High-performance 2D rhenium disulfide (ReS_2) transistors and photodetectors by oxygen plasma treatment. *Adv. Mater.* **28**, 6985–6992 (2016).

16. Q. Zhang, W. Wang, J. Zhang, X. Zhu, Q. Zhang, Y. Zhang, Z. Ren, S. Song, J. Wang, Z. Ying, R. Wang, X. Qiu, T. Peng, L. Fu, Highly efficient photocatalytic hydrogen evolution by ReS_2 via a two-electron catalytic reaction. *Adv. Mater.* **30**, e1707123 (2018).

17. Q. Zhang, L. Fu, Novel insights and perspectives into weakly coupled ReS_2 toward emerging applications. *Chem* **5**, 505–525 (2019).

18. S. Tongay, H. Sahin, C. Ko, A. Luce, W. Fan, K. Liu, J. Zhou, Y.-S. Huang, C.-H. Ho, J. Yan, D. Frank Ogletree, S. Aloni, J. Ji, S. Li, J. Li, F. M. Peeters, J. Wu, Monolayer behaviour in bulk ReS_2 due to electronic and vibrational decoupling. *Nat. Commun.* **5**, 1–6 (2014).

19. J. B. Khurgin, Expanding the photonic palette: Exploring high index materials. *ACS Photonics* **9**, 743–751 (2022).

20. K. Rechcińska, M. Król, R. Mazur, P. Morawiak, R. Mirek, K. Łempicka, W. Bardyszewski, M. Matuszewski, P. Kula, W. Piecek, P. G. Lagoudakis, B. Piętka, J. Szczytko, Engineering spin-orbit synthetic Hamiltonians in liquid-crystal optical cavities. *Science* **366**, 727–730 (2019).

21. E. Altman, L. M. Sieberer, L. Chen, S. Diehl, J. Toner, Two-dimensional superfluidity of exciton polaritons requires strong anisotropy. *Phys. Rev. X* **5**, 011017 (2015).

22. R. Gogna, L. Zhang, H. Deng, Self-hybridized, polarized polaritons in ReS_2 crystals. *ACS Photonics* **7**, 3328–3332 (2020).

23. D. Chakrabarty, A. Dhara, K. Ghosh, A. K. Pattanayak, S. Mukherjee, A. R. Chaudhuri, S. Dhara, Interfacial anisotropic exciton-polariton manifolds in ReS_2. *Optica* **8**, 1488–1494 (2021).

24. A. Dhara, D. Chakrabarty, P. Das, A. K. Pattanayak, S. Paul, S. Mukherjee, S. Dhara, Additional excitonic features and momentum-dark states in ReS_2. *Phys. Rev. B* **102**, 161404 (2020).

25. A. Arora, J. Noky, M. Drüppel, B. Jariwala, T. Deilmann, R. Schneider, R. Schmidt, O. Del Pozo-Zamudio, T. Stiehm, A. Bhattacharya, Highly anisotropic in-plane excitons in atomically thin and bulklike 1 T′-ReSe. *Nano Lett.* **17**, 3202–3207 (2017).

26. C. H. Liang, Y. H. Chan, K.-K. Tiong, Y. S. Huang, Y. M. Chen, D. O. Dumcenco, C. H. Ho, Optical anisotropy of Au-doped ReS_2 crystals. *J. Alloys Compd.* **480**, 94–96 (2009).

27. D. A. Chenet, O. B. Aslan, P. Y. Huang, C. Fan, A. M. Van Der Zande, T. F. Heinz, J. C. Hone, In-plane anisotropy in mono- and few-layer ReS_2 probed by Raman spectroscopy and scanning transmission electron microscopy. *Nano Lett.* **15**, 5667–5672 (2015).

28. O. B. Aslan, D. A. Chenet, A. M. Van Der Zande, J. C. Hone, T. F. Heinz, Linearly polarized excitons in single- and few-layer ReS_2 crystals. *Acs Photonics* **3**, 96–101 (2016).

29. S. Sim, D. Lee, M. Noh, S. Cha, C. H. Soh, J. H. Sung, M.-H. Jo, H. Choi, Selectively tunable optical Stark effect of anisotropic excitons in atomically thin ReS_2. *Nat. Commun.* **7**, 1–6 (2016).

30. J. Jadczak, J. Kutrowska-Girzycka, T. Smoleński, P. Kossacki, Y. Huang, L. Bryja, Exciton binding energy and hydrogenic Rydberg series in layered ReS_2. *Sci. Rep.* **9**, 1–9 (2019).

31. J. Wang, Y. J. Zhou, D. Xiang, S. J. Ng, K. Watanabe, T. Taniguchi, G. Eda, Polarized light-emitting diodes based on anisotropic excitons in few-layer ReS_2. *Adv. Mater.* **32**, 2001890 (2020).

32. H. Deng, H. Haug, Y. Yamamoto, Exciton-polariton bose-einstein condensation. *Rev. Mod. Phys.* **82**, 1489–1537 (2010).

33. J. J. Hopfield, Theory of the contribution of excitons to the complex dielectric constant of crystals. *Phys. Rev.* **112**, 1555–1567 (1958).

34. L. C. Andreani, F. Bassani, A. Quattropani, Longitudinal-transverse splitting in Wannier excitons and polariton states. *Il Nuovo Cimento D* **10**, 1473–1486 (1988).

35. M. M. Denisov, V. P. Makarov, Longitudinal and transverse excitons in semiconductors. *Phys. Status Solidi B* **56**, 9–59 (1973).

36. A. Canales, D. G. Baranov, T. J. Antosiewicz, T. Shegai, Abundance of cavity-free polaritonic states in resonant materials and nanostructures. *J. Chem. Phys.* **154**, 024701 (2021).

37. V. Liu, S. Fan, S4: A free electromagnetic solver for layered periodic structures. *Comput. Phys. Commun.* **183**, 2233–2244 (2012).

38. Q. Cui, J. He, M. Z. Bellus, M. Mirzokarimov, T. Hofmann, H. Chiu, M. Antonik, D. He, Y. Wang, H. Zhao, Transient absorption measurements on anisotropic monolayer ReS_2. *Small* **11**, 5565–5571 (2015).

39. T. Wen, J. Li, Q. Deng, C. Jiao, M. Zhang, S. Wu, L. Lin, W. Huang, J. Xia, Z. Wang, Analyzing anisotropy in 2D rhenium disulfide using dichromatic polarized reflectance. *Small* **18**, 2108028 (2022).

40. J. Gu, V. Walther, L. Waldecker, D. Rhodes, A. Raja, J. C. Hone, T. F. Heinz, S. Kéna-Cohen, T. Pohl, V. M. Menon, Enhanced nonlinear interaction of polaritons via excitonic Rydberg states in monolayer WSe_2. *Nat. Commun.* **12**, 1–7 (2021).

41. T. Yagafarov, D. Sannikov, A. Zasedatelev, K. Georgiou, A. Baranikov, O. Kyriienko, I. Shelykh, L. Gai, Z. Shen, D. Lidzey, P. Lagoudakis, Mechanisms of blueshifts in organic polariton condensates. *Commun. Phys.* **3**, 1–10 (2020).

42. K. Orfanakis, S. K. Rajendran, V. Walther, T. Volz, T. Pohl, H. Ohadi, Rydberg exciton–Polaritons in a Cu_2O microcavity. *Nat. Mater.* **21**, 1–6 (2022).

43. V. Walther, R. Johne, T. Pohl, Giant optical nonlinearities from Rydberg excitons in semiconductor microcavities. *Nat. Commun.* **9**, 1–6 (2018).

44. X. Liu, T. Galfsky, Z. Sun, F. Xia, E. Lin, Y.-H. Lee, S. Kéna-Cohen, V. M. Menon, Strong light–Matter coupling in two-dimensional atomic crystals. *Nat. Photonics* **9**, 30–34 (2015).

45. N. B. Mohamed, K. Shinokita, X. Wang, H. E. Lim, D. Tan, Y. Miyauchi, K. Matsuda, Photoluminescence quantum yields for atomically thin-layered ReS_2: Identification of indirect-bandgap semiconductors. *Appl. Phys. Lett.* **113**, 121112 (2018).

46. Y. Chen, X.-L. Gong, J.-G. Gai, Progress and challenges in transfer of large-area graphene films. *Adv. Sci.* **3**, 1500343 (2016).

47. C. F. Klingshirn, *Semiconductor Optics* (Springer, 2012).
